# Abnormal Position of the Kidney

**Published:** 1898-07

**Authors:** S. J. J. Harger

**Affiliations:** Veterinary Department University of Pennsylvania, Philadelphia, Pa.


### ABNORMAL POSITION OF THE KIDNEY.

By S. J. J. Harger, V.M.D.,
VETERINARY DEPARTMENT UNIVERSITY OF PENNSYLVANIA, PHILADELPHIA, PA.

The subject, an aged gelding, was destroyed for dissecting purposes. The right kidney was normal in shape, but situated on the lateral wall of the pelvis opposite to the neck of the ileum. Its artery was a branch of the external iliac artery, and its vein opened into the pelvic trunk. The ureter, very short, left the posterior extremity of the organ; its termination into the bladder was normal. By rectal examination this organ could have been mistaken for a pathological growth. The left kidney was normal.

